# Prevalence of frailty in people living with HIV over 60 years old in southern Spain

**DOI:** 10.1097/MD.0000000000045282

**Published:** 2026-01-23

**Authors:** Julián Olalla, Yamal Jamal-Ismail, Santiago Vico, Pedro Alarcón, Javier Arenas-Villafranca, Alfonso Del Arco, Javier Pérez-Stachowski, Francisco Rivas-Ruiz, María Dolores Martín-Escalante

**Affiliations:** a Internal Medicine, Universitary Hospital Costa del Sol, Marbella, Spain.

**Keywords:** aging, frailty, HIV, low socioeconomic status, mental disorders

## Abstract

This cross-sectional study aims to describe the prevalence of frailty and pre-frailty in a cohort of people living with HIV in southern Spain. Patients were classified according to Fried frailty phenotype. Questionnaires on anxiety and depression (Hospital Anxiety and Depression scale), loneliness (University of California), resilience (Connor–Davidson Resilience Scale [10-item version]), and socioeconomic information were collected from the participants, their medical history was reviewed to collect comorbidities, non-AIDS events, and drug use. A cluster analysis was conducted to identify participants most likely to be frail. Fifty-two patients were recruited, mostly men, median age 64 years. Four patients were identified as frail (7.7%), 26 as pre-frail (50%), and 22 as robust (43.3%). Frailty was associated with lower socioeconomic level, loneliness, anxiety, depression, and insomnia. Diabetes mellitus was present in all frail patients, and the number of non-antiretroviral medications was higher too. Cluster analysis revealed that the presence of high triglyceride levels and low glomerular filtration rate or high body mass index predicted the presence of frailty. Frailty is associated with certain social and neuropsychiatric characteristics that must be considered. We provide a simple tool to identify those individuals for whom the frailty screening should be started.

## 1. Introduction

Cohorts of people living with HIV (PLHIV) show a progressive increase in their average age, so that it is estimated that in 2030, 70% of these patients will be at least 50 years old.^[1,2]^ When comparing PLWHIV and the HIV-negative population (people without HIV [PWH]), there is evidence of a higher percentage of frail individuals with accumulated comorbidities among PLWHIV.^[3]^

When comparing PLWHIV with PWH, the presence of comorbidities and their number is significantly higher for any decade.^[4]^ In the COBRA cohort,^[5]^ consisting of HIV-infected men aged at least 45 years, undetectable with antiretroviral therapy (ART) and seronegative controls, the difference between chronological and biological age estimated by a series of biomarkers was calculated, yielding an excess of 13 years in PLWHIV. This finding is similar to the excess cardiac age determined in the HOPS cohort, which described an excess cardiac age of 13.7 years among men living with HIV aged 50 to 59 years and 16.4 years among women living with HIV of the same age, when compared with the uninfected population.^[6]^

Frailty is defined as a “biological syndrome of decreased functional reserve and resistance to stressors; due to the cumulative decline of multiple physiological systems resulting in loss of homeostatic capacity and vulnerability to adverse events.”^[7–9]^ Its presence is associated with an increase in adverse events and mortality also in PLWHIV.^[10]^ In PLWHIV, women report worse physical function and quality of life than men.^[11]^

Furthermore, frailty has been associated in HIV patients with a proinflammatory state. In the MACS cohort, it was observed that frail HIV patients had higher C-reactive protein (CRP) levels than non-frail patients, even among virologically suppressed individuals.^[12]^

Different tools can be used to identify frailty in patients with HIV infection, but the main one is the frailty phenotype (FF), designed by Fried et al,^[8]^ measures 5 domains: unintentional weight loss, weakness or decreased hand pressure strength, fatigue, decreased gait speed, and low physical activity. Individuals meeting 1 or 2 of these criteria are classified as pre-frail, while those with 3 or more are classified as frail.

FF is associated in the general population over 65 years of age with the presence of falls, worsening mobility, impaired performance of activities of daily living, hospitalizations, and death at 3 and 7 years,^[8]^ in pre-fragile individuals this association is present, but with less intensity. Also in frail HIV-infected individuals, the risk of death doubles in frail patients compared to non-frail ones^[13]^ and there is an increased risk of hospitalization, AIDS, and non-AIDS events and falls.^[14]^ In particular, in PLWHIV over 40 years of age, the risk of falls is 17 times higher among the frail than among the robust.^[15]^ In addition, several cohort studies have shown that FF is observed at younger ages in HIV-infected patients versus seronegative controls.^[16,17]^ FF is the frailty measurement tool that has been most frequently used in studies on PLHIV and it has been validated in PLWIH and PWH, which allows these populations to be compared using the same measurement tool. The fact that it focuses on 5 different items allows for the selection of specific areas for improvement.

The prevalence of frailty in general population ≥50 years in 22 European countries was 12% in community individuals, increasing with age, female sex, low socioeconomic status, and presence of comorbidities.^[18]^ In cohorts of PLWHIV, with mean ages ranging from 41 to 49 years, the prevalence of pre-frailty is up to 39%, and that of frailty 2% to 9%,^[16]^ a recent meta-analysis analyzing the prevalence of frailty in PLWHIV over 50 years (totaling 6584 patients), found that pre-frailty has a prevalence of 47.2% and frailty 10.9%.^[19]^ We also know that there is a great difference in the prevalence of frailty even within the same country.^[20]^

It is important to identify frailty in order to be able to act on it and return the patient to a more optimal state or, if necessary, to limit the therapeutic effort in cases in which it is considered irreversible.

In this study, we propose to investigate the prevalence of frailty determined by FF in a sample of PLWHIV ≥60 years of age and to explore the factors associated with it in a sample of patients from southern Spain.

## 2. Materials and methods

This is a cross-sectional study carried out in the HIV infection clinic of the Hospital Costa del Sol (Marbella, Spain), which had 189 patients aged at least 60 years in January 2022. Telephone contact was made by the investigators to propose participation in the study. Participation was offered to PLHIV ≥60 years with stable residence in our area. Patients with Barthel < 90, life prognosis < 1 year, diagnosis of child stage C cirrhosis, on renal replacement therapy, with a diagnosis of dementia, bone fracture in the previous 3 months interfering with walking ability or prehensile strength in the judgment of the investigator, diagnosis prior to inclusion in the study of diseases requiring use of chemotherapy, radiotherapy or non-minor surgery were excluded, end of chemotherapy or radiotherapy in the 3 months prior to inclusion in the study, major surgery in the 3 months prior to inclusion in the study, current diagnosis of consumptive syndrome, active neoplasm at the time of inclusion in the study, except for non-melanoma skin cancer or anal carcinoma in situ, or presenting any of the absolute contraindications for the performance of the healthy exercise program (File S1, Supplemental Digital Content, https://links.lww.com/MD/Q388). Approval was obtained from the local Ethics Committee of the Costa del Sol Universitary Hospital.

After signing the informed consent form, sociodemographic and anthropometric data were collected, as well as toxic habits, historical, and current data on HIV infection, presence of comorbidities and hospital admissions, analytical data, presence of polypharmacy (≥5 non-HIV drugs consumed for at least 90 days prior to inclusion in the study). Questionnaires on anxiety and depression (Hospital Anxiety and Depression scale), loneliness (University of California, Los Angeles Loneliness Scale), physical activity (International Physical Activity Questionnaire), adherence to the Mediterranean diet, insomnia severity questionnaire (Insomnia Severity Index), and resilience (Connor–Davidson Resilience Scale [10-item version]) were also completed. FF was determined by classifying patients into robust (no criteria), pre-fragile (1 or 2 criteria), and fragile (≥3 criteria).

A descriptive analysis was performed using measures of central tendency, dispersion and position for quantitative variables, and frequency distribution for qualitative variables; presenting the point estimates of outcome variables with respective 95% confidence intervals. All descriptive analyses of cross-sectional study will be performed for the total sample and segmented by frailty status. IBM SPSS Statistics for Windows, Version 28.0 (IBM Corp., Armonk) was used for the statistical analysis. For all the comparations, level of significance was *P* < .05.

For cluster analysis and with the aim of identifying predictive patterns associated with frailty and differentiating frail patients (frail and pre-frail) from robust ones, an exploratory cluster analysis was performed. Initially, all potentially influential variables on frailty were considered, and a Lasso regression was applied to reduce dimensionality. Subsequently, a decision tree analysis was utilized. The decision tree is an algorithm that uses the values of the most predictive variables to stratify the probability of belonging to the risk group (in this case, frailty), establishing specific cutoff points or rules that distinguish patients with higher and lower risk.

## 3. Results

Fifty-two patients were recruited, 41 of whom were men (78.8%), with a median age of 64 years (AIQ: 4.5). Of the patients, 76.9% were of Spanish origin, followed by patients of Hispanic-American origin (9.6%), from the rest of Europe (7.7%), and from the Maghreb (5.8%). A total of 53.8% of the patients had a stable partner, 73.1% of them owned their own home, and 50% had an income of more than 1500 euros per month. Of the total cohort, only 1.9% did not have at least basic education and 71.2% had completed secondary or higher education. The median body mass index (BMI) was 25.95 kg/m^2^.

Regarding HIV infection, the age of infection was 21 years and the age of antiretroviral treatment was 15 years. The main characteristics of this cohort are shown in Table 1.

**Table 1:** Main characteristics of the study population and comparison according to frailty phenotype.

Variable	Total	Frail (n: 4)	Pre-frail (n: 26)	Robust (n: 22)	*P*
Sex (male)	78.8	25	76.9	90.9	**.012**
Age (yr)	64	63	64	63	ns
Country of origin Spain	76.9	75	76.9	77.3	ns
Marital status					ns
Single	38.5	25	46.2	38.5	
Life as a couple	40.4	25	46.2	40.4	
Divorced	13.5	50	3.8	13.5	
Widow	7.7	0	3.8	7.7	
Children	61.5	100	50	68.2	ns
Homeownership	73.1	50	73.1	77.3	ns
Monthly income (euros/month)					**<.005**
<600	11.5	**75**	0	13.6	
601–1000	26.9	**25**	30.8	22.7	
1001–1500	11.5	0	7.7	18.2	
1501–2000	40.4	0	50	36.4	
>2000	9.6	0	11.5	9.1	
Live in company	65.4	100	53.8	72.7	ns
Education level					**.004**
No studies	1.9	**25**	**0**	**0**	
Primary	26.9	0	30.8	27.3	
Secundary	40.4	75	26.9	50	
Universitary	30.8	**0**	42.3	22.7	
BMI (kg/m^2^)	25.95	25.05	26.55	24.6	ns
Tobacco use					ns
No	34.6	25	42.3	27.3	
Former smoker	30.8	75	23.1	31.8	
Smoker	34.6	0	34.6	40.9	
Alcohol consumption	42.3	0	42.3	50	ns
ChemSex use (%)	5.8	0	3.8	9.1	ns
HIV acquisition route					ns
Heterosexual	48.1	75	50	40.9	
Men who have sex with men	32.7	0	38.5	31.8	
Intravenous drug users	19.2	25	11.5	27.3	
Time from HIV diagnosis (yr)	21	17.5	20	22.5	ns
Time with ART (yr)	15	12	15	15	ns
Number of ART lines (n)	5	7.5	5	6	ns
Basal CD4/CD8 ratio	0.98	0.77	0.96	1.03	ns
Undetectable in the last year	92.3	75	96.2	90.9	ns
HBV coinfection	9.6	0	3.8	18.2	ns
HCV coinfection	32.7	50	26.9	36.4	ns
Diabetes mellitus	26.9	**100**	**30.8**	**9.1**	**.001**
High blood pressure	30.8	25	30.8	31.8	ns
Dyslipemia	67.3	100	69.2	59.1	ns
Obesity	11.5	25	11.5	9.1	ns
Cardiovascular event	26.9	25	30.8	22.7	ns
Cerebrovascular event	3.8	0	3.8	4.5	ns
Cancer	15.4	0	19.2	13.6	ns
HAD depression					**<.0005**
No	88.5	**0**	**92.3**	**100**	
Yes	3.8	**25**	**3.8**	**0**	
Doubtful	7.7	**75**	**3.8**	**0**	
HAD depression score	2	**9.5**	**2.5**	**0.5**	**.002**
HAD anxiety					**.001**
No	73.1	**0**	**69.2**	**90.9**	
Sí	13.5	**75**	**11.5**	**4.5**	
Doubtful	13.5	**25**	**19.2**	**4.5**	
HAD anxiety score	5	**11.5**	**6**	**4**	**.006**
UCLA loneliness scale score	35	**22**	**34.5**	**37**	**.034**
ISI insomnia scale score	6	**22**	**4**	**6**	**.007**
Degree of insomnia ISI scale					**.01**
No insomnia	54.9	**0**	**57.7**	**61.9**	
Mild	21.6	**0**	**23.1**	**23.8**	
Moderate	15.7	**50**	**15.4**	**9.5**	
Severe	7.8	**50**	**3.8**	**4.8**	
CDRISC 10 resilience scale score	32	28	30.5	35	ns
Weekly METs Ipaq questionnaire	5072	**594**	**2812.5**	**8691**	**<.0005**
Number of non-ARV medications	4	**11**	**4**	**2**	**.037**
Use of drugs					
Anti-aggregants	25	25	26.9	22.7	ns
Antidepressants	15.4	**75**	**11.5**	**9.1**	**.003**
Analgesics	28.8	75	30.8	18.2	ns
Statins	61.5	100	61	54.5	ns
PPIs	40.4	75	42.3	31.8	ns
Benzodiazepines	25	**100**	**19.2**	**18.2**	**.001**
Genitourinary tract	15.7	0	11.5	23.8	ns
Polypharmacy	45.1	100	40	40.9	ns
Analytical parameters					
Glucose (mg/mL)	102	114.5	103.5	101	ns
Estimated glomerular filtration rate (mL/min)	73	77	64	76.5	ns
Total bilirubin (mg/mL)	0.7	**0.35**	**0.7**	**0.65**	**.03**
Total cholesterol (mg/mL)	169.5	160.5	171.5	166.5	ns
LDL cholesterol (mg/mL)	104	90	101.5	107	ns
HDL cholesterol (mg/mL)	47.5	46	49	45	ns
Triglycerides (mg/mL)	106	**151**	**119.5**	**88.5**	**.008**
CRP (mg/mL)	3	8.7	3	3	ns
D-dimers (mg/mL)	266.5	733	264	264	ns
Interleukin 6	2.75	5.7	2.7	2.5	ns
Hemoglobin (g/dL)	15.1	14.35	15.1	15.3	ns
Platelets (cells/µL)	229	279.5	228	219	ns

Qualitative variables are expressed as percentages and quantitative variables as median. Bold values indicate statistically significant differences (*P* < .05).

ART = antiretroviral treatment, BMI = body mass index, CDRISC10 = Connor–Davidson Resilience Scale (10-item version), CRP = C-reactive protein, HAD = Hospital Anxiety and Depression scale, HBV = hepatitis B virus, HCV = hepatitis C virus, ISI = Insomnia Severity Index, ns = non significative, PPIs = proton pump inhibitors, UCLA = University of California, Los Angeles Loneliness Scale.

Four patients were identified as frail (7.7%), 26 as pre-frail (50%), and 22 as robust (43.3%). Of the FF criteria, the most frequently met was low physical activity (52.2%), followed by fatigue (15.4%), decreased speed (13.5%), unintentional weight loss (11.5%), and decreased prehensile strength (4%). When comparing the 3 groups of patients according to their phenotype, we observe that, although statistical significance is not reached, there are trends in certain variables. Thus, the proportion of women decreases between robust and frail patients, as can be seen in Table 1. When analyzing monthly income and level of studies, all the frail patients have a monthly income of <1000 euros, and almost all have a monthly income of <600 euros; likewise, no frail university students are identified in our cohort. Regarding toxic habits, the number of pack-years of tobacco consumed throughout life was higher in the frail than in the pre-fragile and robust, while alcohol and chemsex consumption showed the opposite trend. Frail patients had a shorter duration of HIV infection and time on ART, but with a higher number of ART lines, a lower CD4/CD8 ratio and a lower proportion of undetectable. The prevalence of diabetes also showed a gradient, with 100% of the frail patients being diabetic; the prevalence of obesity was also higher in these patients. Among the neuropsychiatric comorbidities, it was observed that according to the Hospital Anxiety and Depression scale score, there were no depressed patients among the robust patients, but the frail patients had depression or suspected depression, with almost the same situation being reproduced in the case of anxiety. Similarly, 75% of the frail patients felt lonely, but this percentage was reduced to 22.7% in the robust patients. The same gradient effect was seen in insomnia, since 100% of the frail patients had moderate or severe insomnia, with percentages of 19.2% and 14.3% among the pre-fragile and robust patients. Taking the frail as a reference, the physical activity referred to by the weekly consumption of metabolic equivalents was almost 6 times higher in the pre-fragile and 14 times higher in the robust.

**Figure 1.**
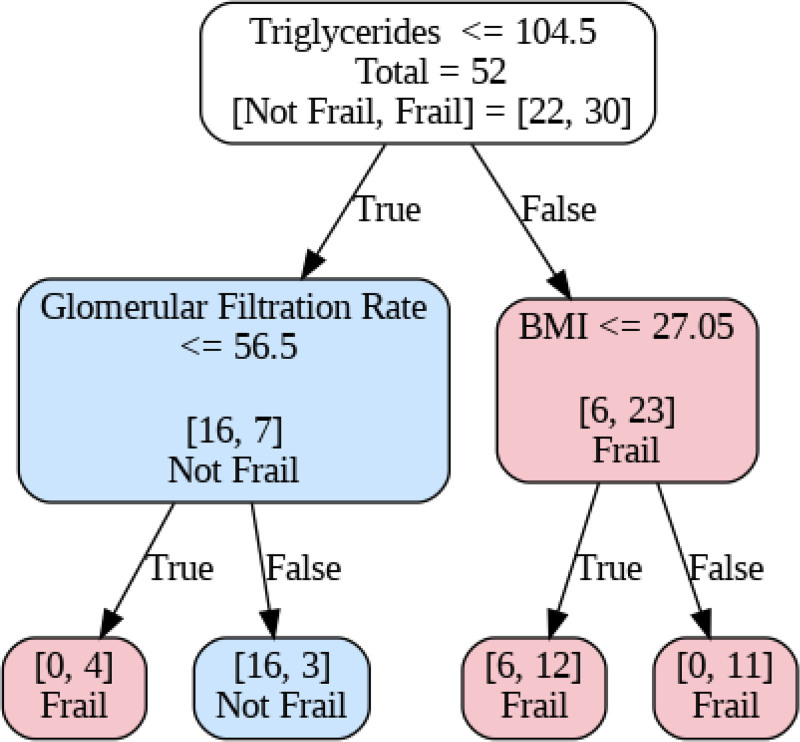
Clustering decision tree.

The number of non-antiretroviral drugs was also higher in frail patients, with 100% of them having polypharmacy. As for the analytical data, it should be noted that triglyceride levels were higher in frail patients. Cluster analysis was performed, with initial selection and Laso analysis, resulting in 12 variables: BMI, triglycerides, estimated glomerular filtration rate, alkaline phosphatase, D-dimer, resilience score (Connor–Davidson Resilience Scale [10-item version]), total cholesterol, basal CD4, CD4/CD8, “living alone,” interleukin 6, and CRP. In this case, the decision tree identified triglycerides as the initial variable of greatest weight (cutoff point at 104.5 mg/dL). Below this value, the probability of not being frail increases, adjusted by the glomerular filtration rate (≤56.5 mL/min), whose decrease increases the risk of frailty. Conversely, above 104.5 mg/dL of triglycerides, the BMI (cutoff at 27.05 kg/m²) refines the classification, indicating a higher probability of frailty for elevated triglyceride values, regardless of BMI (Fig. 1).

## 4. Discussion

In this sample of patients from southern Spain, we have identified a proportion of frail patients of 7.7% and pre-frail patients of 50%. The prevalence of frailty is similar to that reported in different studies in PWH for Spanish population,^[18]^ estimated between 4% and 27% for individuals from the community, but in those cohorts, the age of inclusion in most studies were ≥65 or ≥75 years, while our median age was 64 years. Other cohorts of PLWHIV have however reported higher frailty prevalence, around 15%.^[21,22]^ A Spanish study evaluated 117 PLHIV with a median age of 61.3 years and explored the prevalence of frailty using the FF, finding a frailty prevalence of 15.4%. In that study, the median CD4/CD8 ratio was 0.79, lower than the 0.98 reported in our study. In fact, the value reported in that cohort was similar to that of our subgroup of frail patients. The CD4/CD8 ratio is an independent factor associated with mortality and also with frailty. Admittedly, our cohort excluded patients with a Barthel index < 90, thus including only independent or mildly dependent patients. Although this may mean that the prevalence of frailty is lower than that recorded in other cohorts, it also gives us information on patients in whom we would not a priori presume the presence of frailty or pre-frailty.

Like other studies,^[21]^ we found in ours an association between a low baseline CD4/CD8 ratio and the presence of frailty, this fact, together with a higher number of ART lines despite less time of infection would argue in favor of a higher number of ART interruptions, either due to dropouts, toxicity or failure, although this aspect was not studied. Other studies have shown an association between frailty and AIDS, also revealing a state of CD4 lymphocyte depletion.^[17]^ Although statistical significance was not reached, a gradient of higher US-CRP, IL-6, and D-dimer values is observed, translating persistent inflammatory activation. Previous studies have shown how increased inflammatory activity in the form of higher levels of IL-6 or soluble tumor necrosis factor receptor is linked to a transition to frailty over time.^[23]^ Although antiretroviral treatment is effective, it does not completely reverse inflammatory activity. This proinflammatory state may be implicated in a higher incidence of non-AIDS events, accelerated aging, and the presence of frailty.^[24,25]^

We also evidenced an association between the presence of frailty and socioeconomic status, thus, all the frail had monthly incomes <1000 euros and none were university graduates. They also obtained higher scores on the loneliness questionnaire than the rest of the patients in agreement with studies that have found an association between loneliness and frailty.^[22]^ A recent Spanish study showed an association between loneliness and low educational attainment,^[26]^ thus evidencing a “social frailty” that goes hand in hand with the classic FF, in fact there are studies that link psychosocial stress with the activation of stress response pathways in our organism.^[27]^ Both loneliness and low income are conditions linked to poorer health outcomes,^[28]^ so that, within the approach to frailty, socioeconomic assessment and the fight against loneliness should be considered.

Diabetes was present in all frail patients. Studies of frailty in PLWHIV often focus on the number of comorbidities and the concept of multimorbidity,^[29]^ but there are conditions that occur more frequently in the frail, such as diabetes.^[23,30]^ Regarding the neuropsychiatric sphere, there is a clear association with the presence of anxiety, depression, and insomnia, which occur more frequently and intensely in the frail. In fact, patients identified as frail in our study presented all 3 conditions. Differences were also observed in the number of non-antiretroviral drugs and the presence of polypharmacy. In the context of improving care for our frail patients, a priority line of action should be to review the indication of these medications, their appropriateness and possible deprescription.

Finally, cluster analysis offers us a useful tool when it comes to screening for frailty. Identifying patients with a slight increase in triglycerides and low glomerular filtration rate or BMI > 27 can select the group of individuals for whom to begin the evaluation of FF in the routine clinic. Our study has several limitations. Although the associations found largely reproduce those reported in previous series, in others, such as inflammatory markers, the limited sample size has prevented us from establishing this association. On the other hand, the exclusion of patients with a higher level of dependency may have underestimated the percentage of frail patients, but it gives an idea of the prevalence of frailty in those individuals in whom it is not so evident.

In conclusion, in our study we found association of FF with socioeconomic, neuropsychiatric and immunological sphere, identifying a simple application tool to facilitate frailty screening. It is necessary to identify this condition with a view to addressing specific and multicomponent actions leading to its reversal.

## Acknowledgments

To Dámaris Aguilar and Francisca Ruiz, without whose collaboration and support this project would not have been possible. To all PLHIV who participated in this project, for understanding that their collaboration is fundamental to continue making progress in improving the living conditions of PLHIV.

## Author contributions

**Conceptualization:** Julián Olalla, Pedro Alarcón, Javier Arenas-Villafranca, Alfonso Del Arco, Javier Pérez-Stachowski, Francisco Rivas-Ruiz, María Dolores Martín-Escalante.

**Data curation:** Francisco Rivas-Ruiz.

**Formal analysis:** Francisco Rivas-Ruiz.

**Investigation:** Julián Olalla, Yamal Jamal-Ismail, Santiago Vico, Alfonso Del Arco, Javier Pérez-Stachowski.

**Methodology:** Julián Olalla, Pedro Alarcón, Javier Arenas-Villafranca, Alfonso Del Arco, Javier Pérez-Stachowski, Francisco Rivas-Ruiz, María Dolores Martín-Escalante.

**Project administration:** María Dolores Martín-Escalante.

**Supervision:** Julián Olalla, Yamal Jamal-Ismail, Santiago Vico, Pedro Alarcón, Javier Arenas-Villafranca, Alfonso Del Arco, Javier Pérez-Stachowski, Francisco Rivas-Ruiz, María Dolores Martín-Escalante.

**Validation:** María Dolores Martín-Escalante.

**Writing – original draft:** Julián Olalla.

**Writing – review & editing:** Julián Olalla.

## Supplementary Material


