# Supplementary material for: Prevalence of frailty in people living with HIV over 60 years old in southern Spain
Source: Medicine (Baltimore). 2026 Jan 23;105(4):e45282. doi: 10.1097/MD.0000000000045282 (PMC12851660; doi:10.1097/MD.0000000000045282)
Supplement: Supplementary file 1 [file medi-105-e45282-s001.docx]

**Absolute contraindications for the performance of the healthy exercise program:**

- Recent acute myocardial infarction (within the prior 6 months).

- Recent significant change on resting ECG suggestive of myocardial infarction or another acute cardiac event.

- Severe valvular heart disease.

- Unstable angina.

- Uncontrolled atrial or ventricular arrhythmia.

- Third-degree (complete) atrioventricular block without a pacemaker.

- Acute aortic dissection.

- Severe aortic stenosis.

- Acute (active or suspected) endocarditis, myocarditis, or pericarditis.

- Uncontrolled hypertension (>180/100 mmHg).

- Acute thromboembolic disease.

- Severe heart failure.

- Severe acute respiratory failure.

- Uncontrolled orthostatic hypotension.

- Diabetes mellitus with acute decompensation or uncontrolled hypoglycemia.

- Recent fracture (within the prior month).

- Significant emotional distress (psychosis).

- Any other medical condition that, in the judgment of the treating medical team, precludes the performance of physical activity.
